# Fast Fourier Transformation of Peripheral Venous Pressure Changes More Than Vital Signs with Hemorrhage

**DOI:** 10.1093/milmed/usy303

**Published:** 2019-03-21

**Authors:** Patrick C Bonasso, Melvin S Dassinger, Brady McLaughlin, Jeffrey M Burford, Kevin W Sexton

**Affiliations:** 1 Department of Pediatric Surgery, University of Arkansas for Medical Sciences, 1 Children’s Way, Little Rock, AR; 2 University of Arkansas for Medical Sciences College of Medicine, 4301 W Markham St #550, Little Rock, AR; 3 Department of Surgery, University of Arkansas for Medical Sciences, 4301 W Markham St #550, Little Rock, AR

**Keywords:** hemorrhage, peripheral venous pressure

## Abstract

Vital signs are included in the determination of shock secondary to hemorrhage; however, more granular predictors are needed. We hypothesized that fast Fourier transformation (FFT) would have a greater percent change after hemorrhage than heart rate (HR) or systolic blood pressure (SBP). Using a porcine model, nine 17 kg pigs were hemorrhaged 10% of their calculated blood volume. Peripheral venous pressure waveforms, HR and SBP were collected at baseline and after 10% blood loss. FFT was performed on the peripheral venous pressure waveforms and the peak between 1 and 3 hertz (*f*_1_) corresponded to HR. To normalize values for comparison, percent change was calculated for *f*_1_, SBP, and HR. The mean percent change for *f*_1_ was an 18.8% decrease; SBP was a 3.31% decrease; and HR was a 0.95% increase. Using analysis of variance, FFT at *f*_1_ demonstrates a statistically significant greater change than HR or SBP after loss of 10% of circulating blood volume (*p* = 0.0023). Further work is needed to determine if this could be used in field triage to guide resuscitation.

## INTRODUCTION

Hemorrhage is the leading preventable cause of death in the casualty care setting.^1,2^ Recognition of occult hemorrhage and goal-directed fluid resuscitation has remained elusive resulting in delayed triage and poor management of patients with acute blood loss.^3^ Often, occult bleeding is not recognized until the onset of hemodynamic collapse, especially in healthy patients with good compensatory mechanisms.^4,5^ The current standard, vital sign monitoring, does not detect hemorrhage prior to end-organ damage.^3,6^ Fast Fourier transformation (FFT) is a powerful technique that facilitates analysis of signals in the frequency domain which can detect small changes in waveforms.^7^ For FFT, the algorithm divides signals into frequency components for further analysis. We hypothesized that the FFT of the peripheral venous pressure (PVP) waveforms would have a greater percent change in hemorrhage than heart rate (HR) or systolic blood pressure (SBP).

## METHODS

Under an approved Institutional Animal Care and Use Committee protocol, nine Chester White/American Yorkshire cross pigs weighing 17 kg were pre-anesthetized using a combination of Telazol (tiletamine HCl) 2 mg/kg reconstituted with 2.5 mL Xylazine and 2.5 mL of Ketamine given intramuscularly. The pigs were then intubated with a 5.0 mm cuffed endotracheal tube. External warming pads were placed to maintain body temperature. The ventilator (Hallowell 2000, Pitsfield, MA) was set to volume control ventilation with a tidal volume of 10 mL/kg, positive end-expiratory pressure of 5 cm H_2_O, I:E ratio 1:2, FiO_2_ of 0.88, and respiratory rate of 15 breaths per minute. A pulse oximeter was clipped onto the tongue. A 5-lead electrocardiogram was placed. Anesthesia was maintained with 1% isoflurane (Piramal Enterprises Limited, Andhra Pradesh, India).

Surgical exposure of the femoral vein was obtained and a 5-Fr single lumen umbilical catheter (Footprint medical, San Antonio, TX, USA) was placed and secured for blood withdrawal. Data including PVP waveforms, HR, and SBP were collected at baseline and after hemorrhage.

### PVP Waveform Acquisition

An 18-gauge peripheral intravenous (PIV) catheter was placed in the ventral side of the foreleg after induction of anesthesia. The PIV catheter was connected directly to a pressure transducer (ADInstruments, Colorado Springs, CO, USA) via standard high-pressure tubing. The PIV catheter and pressure transducer were secured to the pig and the operating table. The pressure transducer was zeroed and not utilized for drug delivery during study data acquisition. PVP waveforms were collected using a Powerlab data acquisition system. The waveforms were recorded for 5 minutes prior to hemorrhage and 5 minutes after hemorrhage. PVP waveform data were captured at 1 kHZ to allow adequate sampling to perform spectral analysis of the waveform data.

### Hemorrhage

Baseline HR, SBP, and PVP waveforms were collected. Blood was removed at rate of 25cc/min for a total withdrawal of 136cc of blood or 10% of calculated blood volume.^8^ Total blood withdrawal time was 5.5 minutes. HR, SBP and PVP waveforms were collected after the 10% blood loss.

### Spectral Analysis

The spectral FFT was then performed using LabChart (ADInstruments, Colorado Springs, CO, USA) and the peak between 1 and 3 hertz (*f*_1_) corresponding to HR was selected for analysis. Data were recorded at a sampling rate of 1 kHz necessitating 10 seconds of continuous signal for 8K-FFT. Following FFT, the amplitude of each frequency peak was calculated in LabChart. Data were captured in triplicate for each point used in analysis and analyzed using Prism (GraphPad, La Jolla, CA, USA).^6^ Mean baseline and 10% blood loss values for *f*_1_, SBP, and HR were compared using a standard student’s *T*-test. *p*-values <0.001 were considered significant.

### Calculation of Percent Change

The percent change was calculated for HR, SBP, and *f*_1_ and values were normalized for comparison between the baseline and 10% blood loss. The calculation was:

$$\frac{\left[\text{baseline value}-\text{value after blood loss}\right]}{\left(baseline value\right)}\times 100\%.$$

## RESULTS

### PVP Waveforms

**FIGURE 1.**
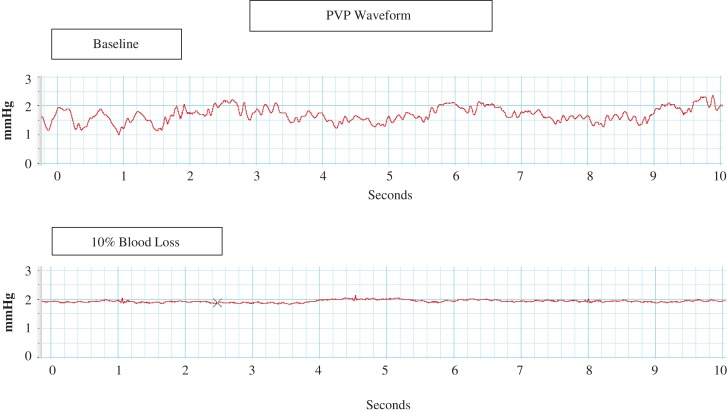
Peripheral venous waveform at baseline and after 10% blood loss.

**FIGURE 2.**
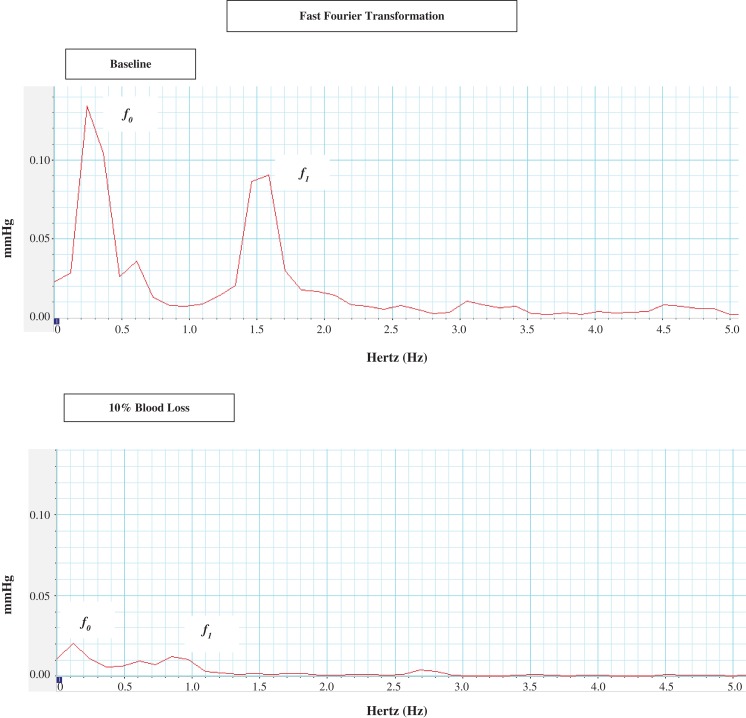
FFT at baseline and after 10% blood loss.

All nine pigs successfully underwent anesthesia, phlebotomy, and PVP waveform data acquisition without complications. Adequate anesthesia was provided with oxygen saturation (SpO_2_) maintained at greater than 97% throughout the experiment. Conversion of the time domain signal into the frequency domain was performed to determine the amplitude of each of the fundamental frequencies recorded during the experiment.^6^ Figure 1 shows a representation of the PVP waveform recorded in LabChart in the time domain both at baseline and after 10% blood volume loss (Fig. 1). FFT at baseline and after 10% loss of circulating blood displays distinct peaks at frequencies *f*_0_ and *f*_1_ (Fig. 2). After FFT, the strongest peak was found at approximately XX Hz, which physiologically corresponds with the animal’s HR. This peak was found in previous studies to correlate most closely with volume status.^6^ The frequency *f*_0_ corresponds to the respiratory rate while *f*_1_ represents the HR, as *f*_1_ signal was strongest and correlate to the porcine model’s HR.^6^ As seen in Figure 2, the *f*_1_ peak pressure is markedly higher for the baseline versus the 10% blood loss group.

### Comparison of *f*_1_, SBP, and HR

Comparisons of mean *f*_1_, SBP, and HR between baseline and after 10% blood were performed using Student’s *T*-test (Table 1). The means at baseline and after 10% blood loss for *f*_1_ were 0.1844 (±0.1076) vs. 0.1496 (±0.0879), respectively (*p* = 0.0023). *f*_1_ was the only significant variable. The mean SBP at baseline and after 10% blood loss was 97.67 (±14.23) vs. 94.44 (±14.21), respectively (*p* = 0.2180). The mean HR at baseline and after 10% blood loss was 146.9 (±5.40) vs. 148.3 (±6.63), respectively (*p* = 0.1590).

**TABLE I.** Comparison of Change for *f*_1_, SBP, and HR

	Baseline	10% Blood Loss	Percentage Change (%)	*p*-Value
*f*_1_	0.1844 ± 0.1076	0.1496 ± 0.0879	18.87	0.0023
Mean ± SD				
**SBP** (mmHg)	97.67 ± 14.23	94.44 ± 14.21	3.31	0.2180
Mean ± SD				
**HR** (beats per min)	146.9 ± 5.40	148.3 ± 6.63	0.95	0.1590
Mean ± SD				

The calculation of percent change for *f*_1_, SBP, and HR was performed. We calculated an 18.8% decrease for *f*_1_, a 3.31% decrease for SBP, and a 0.95% increase for HR.

## DISCUSSION

The main finding of our porcine hemorrhagic animal model is that FFT of PVP waveforms demonstrates greater percent change than HR or SBP after loss of 10% of circulating blood volume.

The potential clinical implications of utilizing a minimally invasive monitor to better predict the amount of hemorrhage as an adjunct to standard vital signs are infinite, since early hemorrhage detection is challenging. We were able to demonstrate significant changes in *f*_1_ during hemorrhage. This model is supported by previous studies demoing hemorrhage detection utilizing PVP waveforms after blood loss.^6,9^ Quantifying the amount of hemorrhage via a non-invasive monitor has potential to diagnosis and treat early shock by identifying hemorrhage prior to changes in vital signs.

Hocking et al proposed that venous waveform analysis could represent a shift from the standard dynamic arterial-based measurements via non-invasive monitoring.^6^ The authors describe the early venous changes via a compensatory mechanism during hemorrhage that may not be identified by other monitoring techniques such as CVP or MAP. Venous constrictions occur in early hemorrhage to increase the cardiac output. Our data support this as the HR and SBP did not have as significant percentage changes as *f*_1_ for 10% of acute blood loss.

To summarize, we further support previous data that show the utility of PVP waveforms in the detection of early hemorrhage prior to standard vital sign changes. The ability to detect early changes has the potential to guide future resuscitation with goal-directed fluid therapy.^6^

## CONCLUSIONS

We investigated the use of peripheral venous waveform analysis as a method to identify hemorrhage compared to standard vital signs of HR and SBP. We determined that the percentage change for *f*_1_ before and after 10% hemorrhage was more sensitive than HR and SBP. Although this is a small animal study, the findings previously noted still need to be validated in future human studies. Further work is needed to determine if this could be a useful field triage criterion to guide resuscitation in both civilian care and military conflict.

## Presentations

Presented as a poster at the 2017 Military Health System Research Symposium.
